# The Teaching of Materia Medica and Therapeutics

**Published:** 1906-12-08

**Authors:** 


### THE TEACHING OF MATERIA MEDICA AND THERAPEUTICS.

The developments which have marked the later history of the science and practice of medicine have, at least to some extent, been associated with a comparative neglect of the art of treatment. There is so much that is exact, confident, and fascinating in modern laboratory methods of diagnosis and in the new triumphs of operative surgery, that the less precise results of medical treatment are apt, especially to the student and junior practitioner, to seem to be unworthy of diligent study. From all quarters come complaints that the careful and scientific prescribing of drugs is a lost art, and that practitioners to a greater and greater extent resort to compound and proprietary preparations which are forced on their attention by the advertisements of the manufacturing druggist. In similar fashion it is repeatedly found that newly qualified men who come fresh from hospital and college to undertake a position as assistant in general practice, though well equipped in modern theories of disease and in the art of physical diagnosis, know little of the art of therapeutics, and are inclined, indeed, to look with suspicion or even contempt on statements proclaiming the remedial value of drugs. An identical state of affairs would also appear to exist on the other side of the Atlantic, for, in the discussion at the recent annual meeting of the British Medical Association, Professor Oliver Osborne, of Yale, indicated that in the State examinations students from all schools stand lower in materia medica and therapeutics than in any other subject. Yet it remains true, to quote another American physician, that the great majority of the sick people in the world must look for help to influences the nature and application of which constitute the subject-matter of the study of therapeutics.

Whatever else may be necessary to promote the more efficient study of therapeutics, it is certain that room must be made for new knowledge and new methods. A detailed knowledge of the natural histories of drugs, of their exact botanical sources, and of the modes of their collection, has no educational interest for the student, and is of no practical help to the practitioner. Exactly the same may be said of a capacity to appraise those physical and chemical characters by which genuine specimens of a drug are of the first importance to those engaged with the quality. Ability and knowledge in these directions are of the first importance to those engaged with the sale of drugs or concerned in the manufacture of alkaloids or other active principles, and it is to the manufacturing chemist and to the pharmacist that the community must look for the discharge of functions of this order. They are not within the province of the medical practitioner, and their details ought not, therefore, to be inflicted on the medical student. Again, there are a considerable number of processes employed in the manufacture of galenical preparations which, though of vital interest to the pharmacist, are of no importance to the student of medicine, and these also might well be removed from the curriculum as practised in our medical schools. Were this proposal enforced, there would be abundant time and opportunity for the demonstration of modern pharmacological results, and also for the consideration of practical therapeutics in relation not only to drugs, but also to diet, hygiene, climate, electricity, and various physical methods of treatment.

In speaking of the necessity of leaving various branches of materia medica in the hands of the pharmacist, it must not, however, be concluded that this means the exclusion of pharmacy from the attention of the medical student. On the contrary, in our judgment at least, the education of the student in this direction needs emphasis. No physician will ever be a skilful prescriber who has not a practical acquaintance with the physical and chemical qualities of the substances he orders for his patients. A course of practical work directed to this end can, we hold, be readily organised for the medical student, and might well be substituted for the present haphazard and uncontrolled discipline by which he is committed for a certain number of hours to the hospital dispensary. Until something of this kind is arranged, it is hopeless to expect the newly qualified practitioner to enter on the construction of well-adjusted and elegant prescriptions. He will be left, as at present, a ready prey to the commercial druggist, and if haply he should escape, this will be due to his personal resolution, and not to his educational equipment.

One further condition of successful instruction in therapeutics may be mentioned. It is that the entire care of this subject shall not be left in the hands of the teacher to whose charge it is officially delegated. Every clinical teacher ought to include in his instruction the study of therapeutics as applied to the cases under his charge, and the student of clinical medicine should be made to realise that all other forms of medical knowledge and skill have this for their justification—the care and treatment of the patient.

